# In Situ Reduced Multi-Core Yolk–Shell Co@C Nanospheres for Broadband Microwave Absorption

**DOI:** 10.3390/ma14164610

**Published:** 2021-08-17

**Authors:** Mu Zhang, Jiahang Qiu, Zhen Xin, Xudong Sun

**Affiliations:** 1 Key Laboratory for Anisotropy and Texture of Materials (Ministry of Education), School of Materials Science and Engineering, Northeastern University, Shenyang 110819, China; 1900581@stu.neu.edu.cn (J.Q.); 2070535@stu.neu.edu.cn (Z.X.); 2 Foshan Graduate School, Northeastern University, Foshan 528311, China

**Keywords:** multi-core yolk–shell, Co@C nanospheres, microwave absorption, high temperature carbonization

## Abstract

The preparation of yolk–shell microwave absorption materials with low density and excellent microwave absorption property requires reasonable design and economical manufacture. In this study, an efficient strategy without any templates or reducing gases has been designed to fabricate multi-core yolk–shell Co@C nanospheres by high temperature carbonization. The results showed that Co_3_O_4_ was completely reduced by the carbon shell to metal cobalt at temperatures above 750 °C. This unique multi-core yolk–shell structure with shell of 600 nm and multiple cores of tens of nanometers can provide sufficient interface and space to reflect and scatter electromagnetic waves. At the same time, the metal cobalt layer and carbon layer provide magnetic loss ability and dielectric loss ability, respectively, making the composite show good wave absorption performance. The minimal RL value of samples carbonized at 750 °C reaches −40 dB and the efficient absorption band reaches 9 GHz with the thickness ranges from 2–9 mm. Therefore, this is a facile, effective and economical strategy to prepare yolk–shell structure, which provides a new idea for the preparation of microwave absorption materials.

## 1. Introduction

With the rapid development of electronic devices, the microwave with a frequency in the 2~18 GHz ranges has become a main part of electromagnetic pollution, so it is urgent to investigate the microwave absorbing materials (MAM) [1,2,3,4]. Among these materials, transition magnetic metals (Fe, Co, Ni) with high specific magnetism, outstanding electric conductivity and low cost has attracted a lot of attention as MAM because of its excellent magnetic loss capability endowed by natural resonance and eddy current effects [5,6,7,8]. However, in order to achieve good impedance matching, so that the electromagnetic wave can incident into the material as much as possible rather than be reflected, it is required that the impedance of the material surface should be as close as possible to the characteristic impedance of free space, that is, the values of relative complex permeability and relative complex dielectric constant should be as close as possible [9,10,11,12]. Hence, the poor impedance matching which causes an undesirable microwave absorption capacity is still a great challenge to transition metals.

In general, it is a common strategy that combines the magnetic loss materials with dielectric loss materials to form core–shell or yolk–shell composites with excellent impedance matching [13,14,15,16]. Yolk–shell structure is a special kind of core–shell structure and the structure can also be called a core@void@shell structure, which means that there is not direct contact between the core and the shell. The existence of void provides unique advantages such as low density and large surface area [17,18,19]. Furthermore, the outside dielectric shell (SnO_2_ [20], MnO_2_ [21], carbon [22]) cannot only effectively protect naked magnetic core from oxidation, but also provide dielectric loss capacity. For instance, Liu and his coworkers [23] have fabricated yolk–shell CoNi@air@TiO_2_ nanospheres with minimum RL value of −41 dB and efficient absorption bandwidth (EABD) of 8 GHz when thickness is 2.5 mm, which have both strong magnetic loss and dielectric loss capacity. Wang et al. [24] synthesized MOF derived multi-interfacial Ni@C@ZnO composites as outstanding MAM with minimum RL value of −55.8 dB and EABD of 4.1 GHz when thickness is 2.5 mm. The ordinary design strategies to form yolk–shell structures use SiO_2_ as a template. Typically, a layer of SiO_2_ was grown on the core using the stöber method, and then the required shell was grown on the SiO_2_ layer. Finally, an alkali solution or hydrofluoric acid was used to remove SiO_2_ to form a void, and the yolk–shell structure was prepared. Nevertheless, the ordinary design strategies with SiO_2_ as a template to form a yolk–shell structure are often too complicated and wasteful. At the same time, the alkaline solution or hydrofluoric acid solution used to etch SiO_2_ layer has certain risks and may react with other core or shell materials, These factors all restrict the development of template method [25,26,27,28].

To address these issues, many strategies to form yolk–shell structures without a template have been developed, such as one-pot [29,30], Ostwald ripening [31], MOF [32,33], Kirkendall effect [34,35] and so on [36,37]. For example, Qin et al. [38] synthesized multi-shelled hollow NiCo_2_O_4_ spheres using one-pot and the minimum Reflection Loss (RL) reached −36.3 dB at 13.16 GHz while the matching thickness is 1.86 mm. Zhao and his groups [39] fabricated yolk–shell Ni@void@SnO_2_ microspheres via the Kirkendall effect and the composites have a minimum RL value of −45.5 dB and EABD of 8.2 GHz when thickness is 2.0 mm. Shi et al. [40] designed Fe@SiO_2_@C-Ni yolk–shell nanospheres in the reduction of high temperature hydrogen gas and the triple-shelled architecture composed of magnetic loss materials and dielectric loss materials endows it with a broad efficient absorption band reaching 8.2 GHz with a thickness of only 2 mm. So far, these template-free methods for preparing yolk–shell structures have achieved the purpose of simple and economical process to some extent, but they still have disadvantages such as uncontrollable size, environmental harm and certain risks, which restrict the further development of the yolk–shell structure [41,42,43].

Herein, considering that the preparation of Co_3_O_4_ and carbon shell is simple and controllable, and carbon can reduce Co_3_O_4_ to metal cobalt at low temperature without by-products a facile strategy was proposed for preparing certain sized yolk–shell Co@C nanospheres without employing templates or dangerous reducing gases. By increasing the temperature of heat treatment, the inner Co_3_O_4_ core was reduced by the carbon shell which is prepared from the carbonization of the outer phenolic resin, and the special structure of the multi-core yolk–shell Co@C nanospheres was obtained. Due to the synergistic effect of the magnetic loss capacity of the multi-cobalt core and the dielectric loss capacity of the outer carbon layer, as well as the multi-scattering attenuation effect of the hollow structure on the electromagnetic wave, the composites exhibit a good microwave absorbing performance. The minimal RL value reaches −40 dB and the EABD reaches 9 GHz with the thickness range of 2–9 mm. This facile method might provide help for the development of high performance MAM.

## 2. Materials and Methods

### 2.1. Materials

All reagents applied in this experiment were analytical grade. Cobaltous nitrate hexahydrate (Co(NO_3_)_3_·6H_2_O), isopropanol (C_3_H_8_O), glycerol (C_3_H_8_O_3_), formaldehyde (37%), resorcinol, ammonia solution (NH_3_OH, 26%), and ethanol were purchased from Sinopharm Chemical Reagent, Co. Ltd. (Shenyang, Liaoning, China). Deionized water used in all experiments was obtained from a Milli-Q system (Millipore, Bedford, MA, USA).

### 2.2. Synthesis of Co_3_O_4_ Nanospheres

Co_3_O_4_ nanospheres were obtained using the method reported by previous literature [44]. In short, 0.272 g of (Co(NO_3_)_3_·6H_2_O) and 10 mL glycerol were dissolved into 50 mL isopropanol to form a homogeneous pink solution under constant stirring. Subsequently, the solution was transferred into a Teflon-lined stainless steel autoclave and kept at 180 °C for 2 h. After cooling to room temperature, the precipitate was collected by centrifugation, washed by deionized water and ethanol several times and then dried at 60 °C for 12 h. The well-dried precursor was then calcined at 350 °C in air for 2 h with a heating rate of 2 °C/min to obtain Co_3_O_4_ nanospheres.

### 2.3. Synthesis of Co@C Nanospheres

The yolk–shell Co@C nanospheres were prepared by a typical in situ polymerization and high-temperature carbonization method [45,46]. Briefly, 0.3 g of synthesized Co_3_O_4_ nanospheres were dispersed in a mixed solution which contains 80 mL of water, 32 mL of ethanol, and 0.4 mL of ammonia. Then, the mixed solution was treated with ultrasonication for 30 min to form a homogeneous suspension. The mixture was then mechanically stirred for 30 min before adding 0.20 g of resorcinol. After resorcinol was completely dissolved, 0.28 mL of formaldehyde solution (37 wt.%) was introduced into the suspension with continuous stirring for 24 h. The suspending Co_3_O_4_@phenolic resin (Co_3_O_4_@PR) nanospheres were separated by centrifugation, and washed by deionized water and ethanol 3 times, respectively. Then, the composites were dried at 60 °C for 12 h. Finally, the Co_3_O_4_@PR nanospheres were carbonized and reduced in a tubular furnace under the atmosphere of argon for 1 h at a heating rate of 5 °C/min. The samples were marked as S1, S2, S3, samples of which were treated under different temperature of 650 °C, 750 °C, 850 °C, respectively.

### 2.4. Characterization

The Crystalline structures and phase composition of obtained products were characterized by X-ray diffraction (XRD, smartlab9) with a Cu Kα source (40 KV, 200 mA). The morphology and size were obtained by scanning electron microscopy (SEM, JSM-7001F) with an accelerating voltage of 20 kV. The transmission electron microscopy (TEM) images were characterized on JEM-2100F with an accelerating voltage of 200 kV. The magnetic hysteresis loops were measured by vibrating sample magnetometer (VSM, Lake Shore Cryotronics, Westerville, OH, USA) at room temperature. The X-ray photoelectron spectrometer (XPS, Axis Supra) was measured on an Axis Supra with an Al Kα X-ray source. The relative complex permeability and relative permittivity of the sample in the frequency range of 2–18 GHz were characterized by a vector network analyzer (VNA, Agilent N5234A). After mixing the products with the paraffin in the ratio of 3:7 uniformly, the annular sample with outer diameter of 7 mm and inner diameter of 3 mm was pressed, and the thickness was about 2.00 mm.

## 3. Results and Discussion

**Figure 1:**
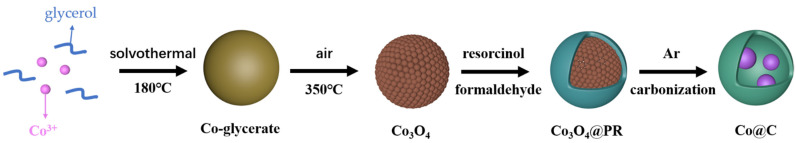
Schematic illustration of the synthetic strategy for multi-core yolk–shell Co@C nanospheres.

The synthesis process of yolk–shell Co@C nanospheres is schematically illustrated in Figure 1. Co-glycerate nanospheres are formed by solvothermal action of Co^3+^ and glycerol, and are then calcined in air to form Co_3_O_4_. Then, resorcinol and formaldehyde formed a phenolic resin layer on the surface of Co_3_O_4_ by in situ polymerization. Finally, the microspheres were carbonized and reduced in protective gas at high temperature to form a multi-core yolk–shell Co@C structure.

**Figure 2:**
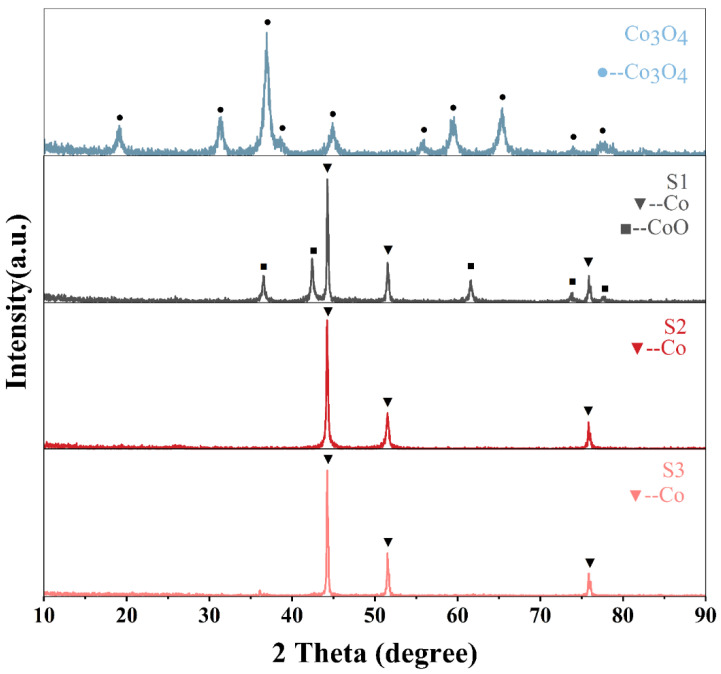
The XRD patterns of Co_3_O_4_, S1, S2, S3.

XRD patterns of Co_3_O_4_, S1, S2 and S3 are shown in Figure 2. Co_3_O_4_ nanospheres’ diffraction peaks can well match the spinel phase of Co_3_O_4_ (JCPDS 73-1701), indicating that the precursor transformed into the Co_3_O_4_ phase after being calcined. The same diffraction peaks at 44°, 51°, 76° in all of the samples can be in good agreement with (111), (200), and (220) planes of fcc metallic cobalt (JCPDS 89-7093), and the remaining diffraction peaks of S1 are matched with that of CoO (JCPDS 43-1004), which indicates that the calcination temperature of 650 °C is not enough to help the carbon layer completely reduce Co_3_O_4_ to zero-valent metallic cobalt. At the same time, although the formed metal cobalt can catalyze the transition from amorphous carbon layer to graphite, the diffraction peak of graphite carbon does not appear in the XRD patterns [47,48].

**Figure 3:**
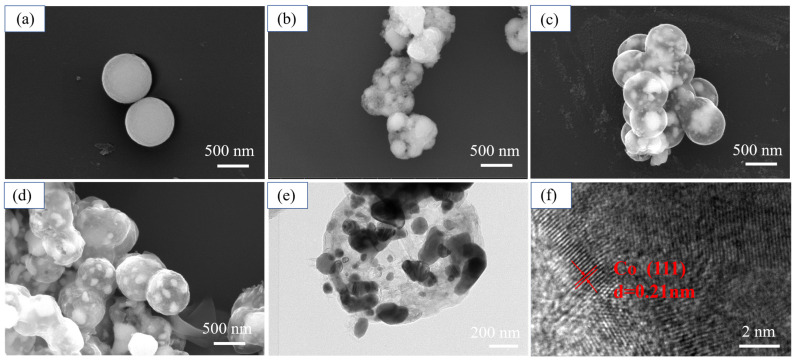
FE-SEM images of (**a**) Co_3_O_4_, (**b**) S1, (**c**) S2, (**d**) S3. TEM image of (**e**) S2. (**f**) HRTEM image of Co wrapped in carbon shell of S2.

Figure 3 illustrates the morphology evolution of Co_3_O_4_ to yolk–shell Co@C nanospheres at different calcination temperatures. Co_3_O_4_ nanospheres are initially synthesized with an average size of 700 nm (Figure 3a). After in situ polymerization and calcination at 650 °C for 1 h, the shape of the nanoparticles is distorted and the internal particles shrink to form several nanospheres of different sizes. Combined with the XRD patterns, it is found that the inner spheres of the carbon layer are composed of metal cobalt and CoO. Compared with the sample calcinated at 650 °C, the outer carbon layer is preserved better in spherical shape at 750 and 850 °C, and the inner cobalt oxide is completely reduced to several irregular metal cobalt particles with similar size (Figure 3c,d). The morphology and structure of the S2 is further characterized by TEM. As can be clearly seen from the image, there are many small particles of tens of nanometers evenly distributed in the inner space wrapped by the carbon layer (Figure 3e). HRTEM images show that the lattice fringes of the internal small particle can match well with the (111) crystal plane of metal cobalt, further proving that cobalt particles are formed after in situ reduction of Co_3_O_4_ in the carbon shell (Figure 3f).

**Figure 4:**
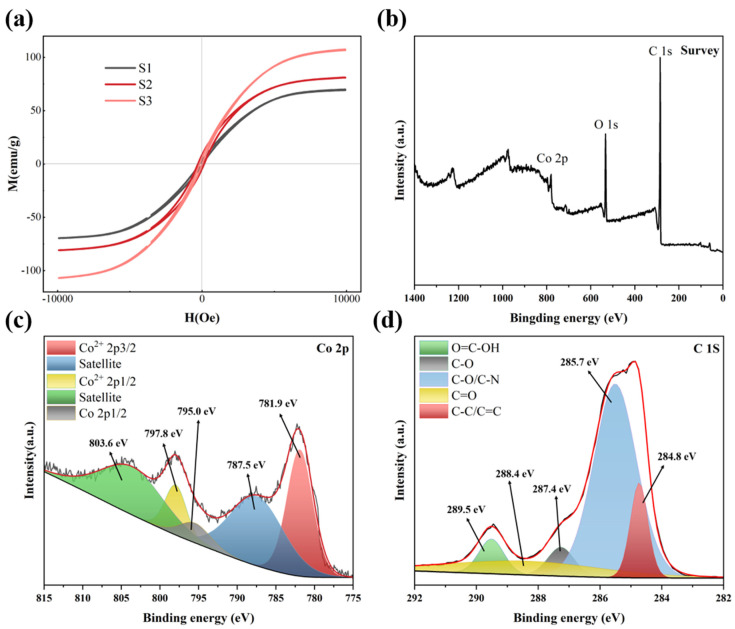
(**a**) Magnetic hysteresis loops of S1, S2 and S3, (**b**) XPS survey spectrum of S2, (**c**) high resolution Co 2p regions of S2, (**d**) high resolution C 1s regions of S2.

The magnetization properties of all the samples are characterized by VSM with a magnetic field of −10,000 Oe–10,000 Oe at room temperature. Figure 4a illustrates the magnetic hysteresis loops of S1, S2 and S3. The saturation magnetization (M_s_) of S1, S2, S3 are 67.7, 81.0, 107.2 emu/g, respectively. It can be found that the M_s_ of the sample increases with the increase in the reduction temperature. The increase in M_s_ from S1 to S2 can be attributed to the enhancement of magnetism caused by the reduction of more antiferromagnet Co_3_O_4_ to ferromagnetic cobalt metal, while the increase in M_s_ from S2 to S3 may be due to the increase in the crystallinity of cobalt metal with the increase in reduction temperature. The coercivity (H_c_) values of S1, S2, S3 are 98.1, 210.3, 90.9 Oe, respectively. For ferromagnetic MAM, initial permeability (μ_i_) can be expressed by the following formula [24,49]:

(1) $$\mu_{i}=\frac{M_{s}^{2}}{{\mathrm{akH}}_{c}M_{s}+b\lambda \xi }$$

where a and b are two constants determined by the material composition, k is the magnetostriction constant, λ is the magnetostriction constant, and ξ is an elastic strain parameter of the crystal. An increase in μ_i_ usually means an increase in magnetic loss capacity, and both high M_S_ and low H_c_ are beneficial to the increase in μ_i_.

In order to further elucidate the surface element composition and atomic configuration of S2, X-ray photoelectron spectroscopy (XPS) was characterized in Figure 4b–d. As can be seen from Figure 4b, the survey spectrum of S2 contains C 1s, Co 2p and O 1s. Figure 2 exhibits high resolution Co 2p spectrum, where the peaks at 781.9 eV and 797.6 eV are attributed to Co^2+^ 2p_3/2_ and Co^2+^ 2p_1/2_ spectrums, respectively [50,51]. Two peaks at 785.5 eV and 803.6 eV are the satellite peaks that accompany the two peaks of Co^2+^. The peak located at 795.0 eV can be assigned to Co^0^. Combined with the XRD spectrum of S2, it can be inferred that the peaks of Co^2+^ are due to the oxidation of surface of sample. The C 1s peaks at 284.8, 285.7, 287.4, 288.4, 289.5 eV are assigned to aromatic ring structure, phenolic hydroxyl group, C-O linkage, carbonyl groups, carbonyl groups, respectively (Figure 4d). It is noteworthy that there is a peak that can be assigned to O 1s in the survey spectrum. According to the XRD result of S2, Co_3_O_4_ was completely reduced to metal cobalt, so it was speculated that the occurrence of the O 1s peak might be due to the incomplete carbonization of phenolic resin and the oxidation of the surface of metal cobalt.

**Figure 5:**
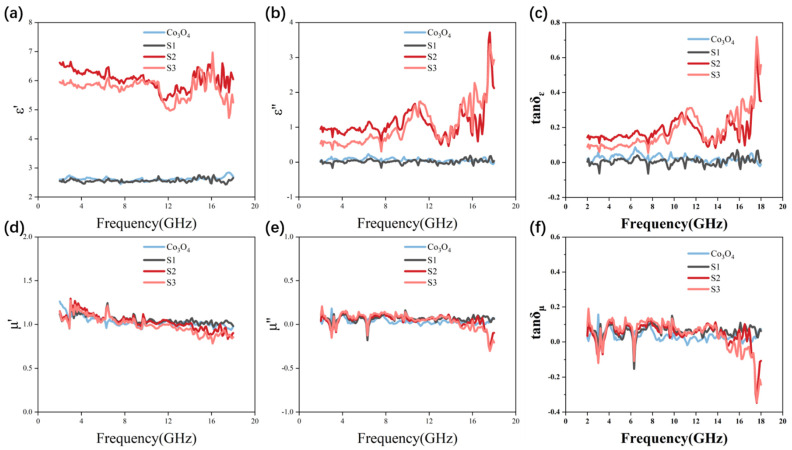
Electromagnetic parameters of Co_3_O_4_ S1 S2, S3: (**a**) ε′, (**b**) ε″, (**c**) dielectric tangent loss factor tanδε, (**d**) μ′, (**e**) μ″, (**f**) magnetic loss tangent factor tanδ_μ_.

Complex permittivity ($\varepsilon_{r}=\varepsilon^{\prime }\;-\;{j\varepsilon }^{\prime \prime }$) and complex permeability ($\mu_{r}=\mu^{\prime }-\;{j\mu }^{\prime \prime }$) of the MAM are measured by the vector network analyzer, which determines the absorbing performance of the MAM at the GHz frequency band (Figure 5a–f). The real parts of complex permittivity (ε′) and complex permeability (μ′) decide the storage capacity of electromagnetic energy, while the imaginary parts of complex permittivity (ε″) and complex permeability (μ″) represent the loss capacity of electromagnetic energy. Figure 5a shows the ε′ of all samples in the range of 2–18 GHz; it can be found that the ε′ of Co_3_O_4_ and S1 are both around 2.5, and there is no change with the increase in frequency. The ε′ of S2 and S3 are relatively high and fluctuate around 6 as the frequency changes. Similarly, the ε″ values of Co_3_O_4_ and S1 fluctuate around 0 with frequency changes, while the ε″ values of S2 and S3 fluctuate around 1 with frequency increasing, with a peak value of around 3.5 at 17.5 GHz (Figure 5b). According to the free electron theory, ε″ ≈ 1/2πρfε_o_ [52,53], where ρ stands for resistivity. It can be found that high conductivity can lead to the increase in ε″. Therefore, the increase in ε″ value of S2 and S3 may be due to the increase in conductivity caused by the increase in metal cobalt content and the deepening of graphitization degree of carbon layer. Dielectric tangent loss factors (tanδ_ε_ = ε″/ε′) represents the dielectric loss capacity of a MAM. The tanδ_ε_ of S2 and S3 varies around 0.2 and reaches the peak value of 0.7 at 17.5 GHz, while the values of Co_3_O_4_ and S1 stay at about 0 with the change in frequency, which proves that the dielectric loss capacity of S2 and S3 is stronger than that of Co_3_O_4_ and S1 in the frequency range of 2–18 GHz (Figure 5c). In terms of complex permeability, the values of both the real part and the imaginary part of the four samples are very close. The real part stays at about 1 with the increase in frequency, while the imaginary part stays at about 0 (Figure 5d,e). Similarly, the magnetic loss tangent factors (tanδμ = μ″/μ′) of the four samples are also very close, and stay at about 0 with the change in frequency, which proves that the four samples only have very weak magnetic loss ability close to each other, and the increase in saturation magnetization does not bring substantial enhancement of magnetic loss ability (Figure 5f).

**Figure 6:**
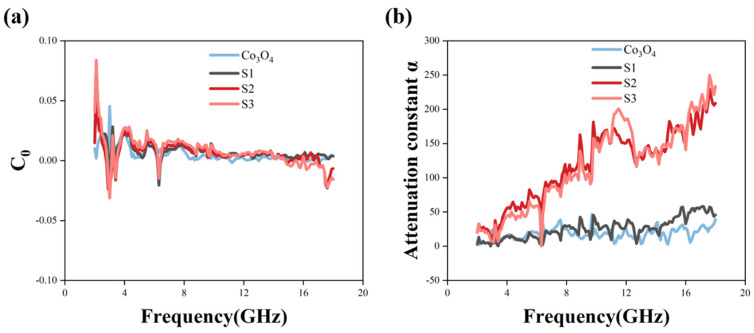
Electromagnetic parameters: (**a**) C_0_ value (**b**) attenuation constant α.

For magnetic microwave absorbing materials, the mechanisms of magnetic loss mainly include natural resonance, eddy current loss, hysteresis loss and domain wall resonance [54,55]. The hysteresis loss can be ignored in the case of a weak electromagnetic field, while the domain wall resonance has a good response behavior, mainly in the MHz frequency band. Therefore, the main mechanisms in the GHz frequency band are natural resonance and eddy current loss. The eddy current loss can be expressed by the following formula [56]:

(2) $$\mu^{\prime \prime }{=2\pi \mu }_{0}{\left(\mu^{\prime }\right)}^{2}\sigma \cdot d^{2}f/3$$

where σ is the electrical conductivity and μ_0_ is the permeability in vacuum. The formula can be converted to $C_{0}=\mu {\left(\mu^{\prime }\right)}^{-2}f^{-1}$, which means that C_0_ will remain constant if the magnetic loss of the material is caused solely by eddy current loss. Figure 6a shows the curve of C_0_ of the samples changing with frequency in the range of 2–18 GHz. When the frequency is in the range of 6–18 GHz, the C_0_ values of the four samples remain around 0 as the frequency changes, which proves that the magnetic loss of the four samples in this frequency band is completely caused by the eddy current loss. Meanwhile, according to the previous report, natural resonance generally occurs in the frequency band below 6 GHz. As shown in Figure 6a, there are certain resonance peaks in the C_0_ values of the four samples in the frequency band between 2–6 GHz. Therefore, it can be speculated that these resonance peaks are caused by natural resonance.

According to the transmission line theory, the attenuation constant α determines the ability of the MAM to attenuate the incident electromagnetic waves, which can be defined as [57]:

(3) $$\alpha =\left(\frac{\sqrt{2}\pi f}{c}\right)\sqrt{\mu_{r}^{\prime \prime }\varepsilon_{r}^{\prime \prime }-\mu_{r}^{\prime }\varepsilon_{r}^{\prime }}+\sqrt{{\left(\mu_{r}^{\prime \prime }\varepsilon_{r}^{\prime \prime }-\mu_{r}^{\prime }\varepsilon_{r}^{\prime }\right)}^{2}+(\mu_{r}^{\prime \prime }\varepsilon_{r}^{\prime }-\mu_{r}^{\prime }\varepsilon_{r}^{\prime \prime }{)}^{2}}$$

where f is the frequency of microwave and c is the velocity of electromagnetic wave in vacuum. As demonstrated in Figure 6b, the α values of the four samples have different increasing trends with the increase in frequency. Among them, the α values of S2 and S3 have a more obvious increasing trend, which can reach 240 at 18 GHz, while the α values of Co_3_O_4_ and S1 can only reach 25 at 18 GHz. Obviously, in the frequency band of 2–18 GHz, the ability of samples S2 and S3 to attenuate electromagnetic waves is much higher than that of Co_3_O_4_ and S1. Such a result is consistent with the results of dielectric loss factor and magnetic loss factor in Figure 5.

**Figure 7:**
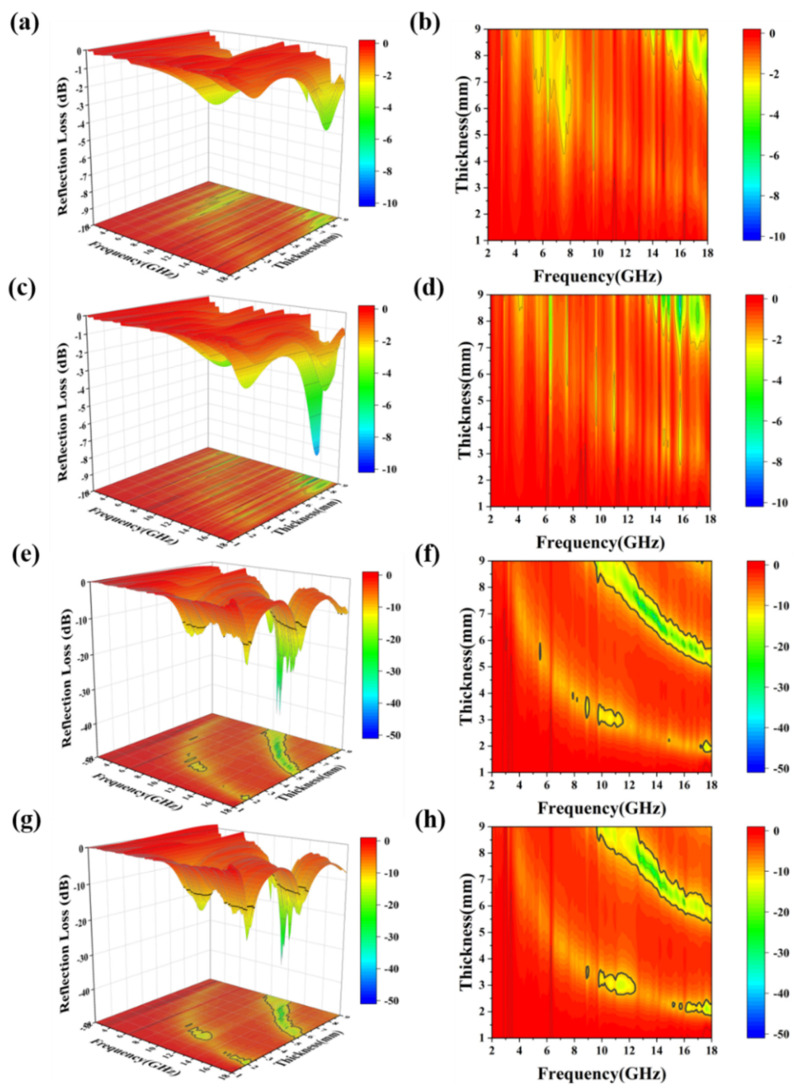
Three-dimensional diagrams and contour diagrams of the RL values of (**a**,**b**) Co_3_O_4_, (**c**,**d**) S1, (**e**,**f**) S2 and (**g**,**h**) S3.

**Figure 8:**
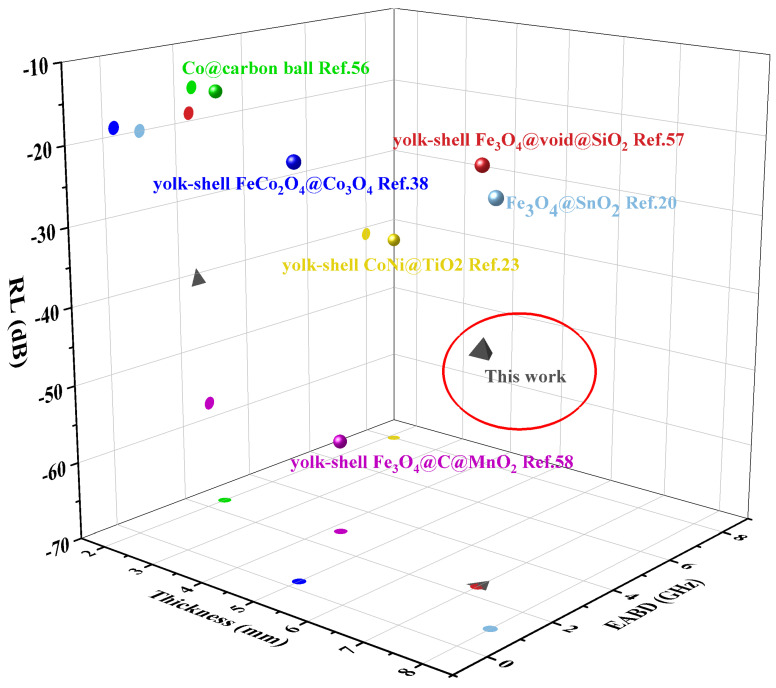
Microwave absorption performance of yolk–shell Co@C composites compared with that of other reported yolk–shell composites.

Based on the complex permittivity and complex permeability of MAM, the reflection loss (RL) can be calculated by the transmission line theory. The calculation method is shown as follows [58,59]:

(4) $$\mathrm{RL}\left(\mathrm{dB}\right)\;=\;20\log\left|\frac{Z_{\mathrm{in}}-1}{Z_{\mathrm{in}}+1}\right|$$

where Z_in_ represents the input impedance of the material, and the calculation formula is as follows:

(5) $$Z_{\mathrm{in}}=\sqrt{\frac{\mu_{r}}{\varepsilon_{r}}}\tanh\left[j\left(\frac{2\pi }{c}\right)\mathrm{fd}\sqrt{\mu_{r}\varepsilon_{r}}\right]$$

where c represents the speed of light in free space, f represents the frequency of electromagnetic waves, and d represents the thickness of the absorber. Figure 6 shows the three-dimensional diagrams and contour diagrams of the RL values of Co_3_O_4_, S1, S2 and S3 in the frequency range of 2–18 GHz and d in the range of 1–9 mm. It can be seen from the figure that Co_3_O_4_ and S1 do not show distinct microwave absorption performance (Figure 7a–d). When the range of the thickness of the absorber is 1–9 mm and the range of frequency of microwave is 2–18 GHz, no RL values reach effective value of −10 dB (absorbing 90% electromagnetic waves). The minimum RL value of Co_3_O_4_ is −4.4 dB when d is 7.6 mm and f is 18 GHz and, the minimum RL value of S1 is −7.4 dB when d is 8.9 mm and f is 15.8 GHz. The performance of such weak absorbing properties is consistent with their poor values of dielectric and magnetic loss factors. In contrast, S2 and S3 both have good wave absorption performance. When the d of S2 is 7.4 mm, the minimum RL value reaches −40 dB (at 13 GHz) and the EAB also reaches 2 GHz (from 11.9 GHZ to 13.9 GHz), while when the d of S3 is 7.9 mm, the minimum RL value is −36 dB and the EAB is 2.2 GHz (from 11.5 GHz to 13.7 GHz). In general, materials with high microwave absorption performance are requested to possess low absorber thickness, low density, wide efficient absorption bandwidth and strong absorption ability. According to Figure 8, compared with other reported yolk–shell structure composites, yolk–shell Co@C nanospheres have good absorption ability and efficient absorption bandwidth, but they need a high absorber thickness, which limits their practical application ability. However, considering the development potential of low-density yolk–shell materials in the field of microwave absorption materials, this simple and controllable method of in situ reduction can be extended to other metal materials to prepare the yolk–shell materials with better microwave absorption properties.

**Figure 9:**
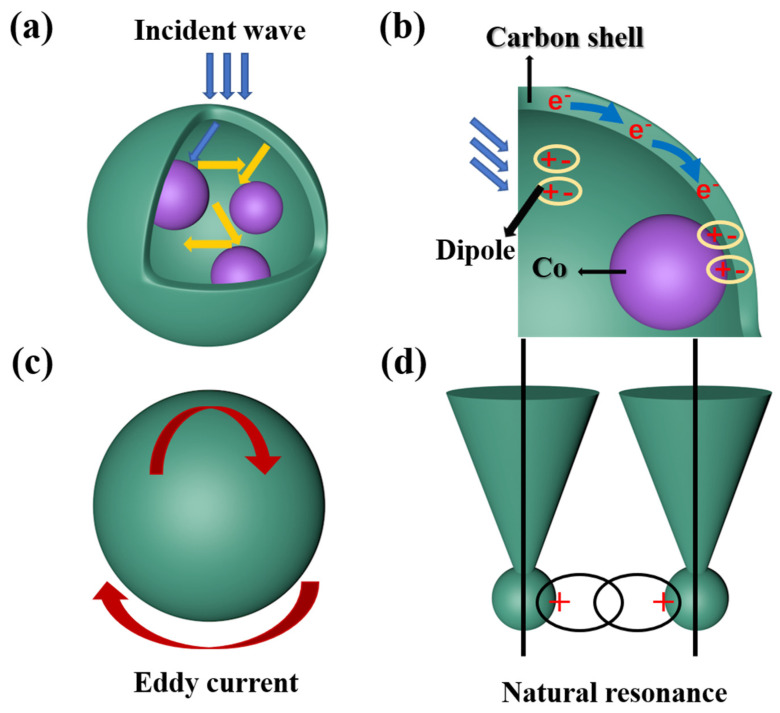
Schematic illustration of EM wave absorption mechanisms for Co@C nanospheres: (**a**) multiple reflection; (**b**) dielectric loss mechanisms; (**c**) eddy current; (**d**) natural resonance.

The microwave absorption performance can be due to the unique yolk–shell structure and the composite loss mechanism of metal cobalt and carbon shell. The microwave absorption mechanism of Co@C nanospheres is shown in Figure 9. First of all, the void space of the multi-core yolk–shell Co@C nanospheres could cause multiple reflection and scattering of electromagnetic wave, resulting in the absorption and exhaustion of incident microwave. Second, as shown in Figure 9b, there are abundant interfaces between multiple metal cobalt cores and carbon layers. Due to the difference in electrical conductivity, a large number of positive and negative charges will be accumulated at these interfaces. In the high-frequency electromagnetic environment, the accumulated charges at these interfaces will generate strong polarization and promote the attenuation of electromagnetic wave. Third, a large number of dipoles caused by defects in the amorphous carbon layer will produce strong relaxation polarization under the action of high frequency external electromagnetic field, which enhances the dielectric loss ability of the composite absorption material. Fourth, with the increase in reduction temperature, the degree of graphitization of the carbon layer increases, and a large number of electrons in the carbon layer can generate micro-current under the action of the external electromagnetic field, which is conducive to enhancing the loss of conductance and converting electromagnetic energy into heat energy. Lastly, natural resonance and eddy current loss will be caused by magnetic metal cobalt under the action of electromagnetic wave in the GHz band, so the electromagnetic wave can be reduced by magnetic loss. These mechanisms above work together to provide excellent microwave absorption performance of Co@C nanospheres.

## 4. Conclusions

In summary, in this study, hollow Co@C yolk–shell nanospheres with multiple metal cores were prepared by in situ reduction of Co_3_O_4_ in the carbon shell. This special structural design can not only reflect and scatter electromagnetic waves multiple times to achieve the purpose of the electromagnetic wave energy dissipation, but also can combine the dielectric loss and magnetic loss, making the composite Co@C absorption materials exhibt excellent absorption performance: the minimal RL value of samples carbonized at 750 °C reaches −40 dB and the EABD reaches 9 GHz with the thickness ranges from 2–9 mm. This unique microstructural design scheme provides a new idea for the preparation of new MAM.

## Data Availability

The data presented in this study are available on request from the corresponding author. The data are not publicly available due to funder data retention policies.
